# Artificial Tumor Microenvironments in Neuroblastoma

**DOI:** 10.3390/cancers13071629

**Published:** 2021-04-01

**Authors:** Colin H. Quinn, Andee M. Beierle, Elizabeth A. Beierle

**Affiliations:** 1 Division of Pediatric Surgery, Department of Surgery, University of Alabama at Birmingham, Birmingham, AL 35205, USA; chquinn@uab.edu; 2 Division of Radiation Oncology, University of Alabama at Birmingham, Birmingham, AL 35205, USA; abeierle@uab.edu

**Keywords:** neuroblastoma, tumor microenvironment, three-dimensional bioprinting, three-dimensional modeling, cancer associated fibroblasts, mesenchymal stromal cells, tumor associated macrophages

## Abstract

**Simple Summary:**

Children with high-risk neuroblastoma have limited therapeutic options poor survival rates. The neuroblastoma tumor microenvironment contributes the lack of response to many interventions so innovative methods are needed to study the effects of the tumor microenvironment on new therapies. In this manuscript, we review the current literature related to the components of the tumor microenvironment and to the use of three-dimensional printing as modality to study cancer. This review highlights the potential for using three-dimensional printing to create an artificial tumor microenvironment in the presence of neuroblastoma to provide improved preclinical testing of novel therapies.

**Abstract:**

In the quest to advance neuroblastoma therapeutics, there is a need to have a deeper understanding of the tumor microenvironment (TME). From extracellular matrix proteins to tumor associated macrophages, the TME is a robust and diverse network functioning in symbiosis with the solid tumor. Herein, we review the major components of the TME including the extracellular matrix, cytokines, immune cells, and vasculature that support a more aggressive neuroblastoma phenotype and encumber current therapeutic interventions. Contemporary treatments for neuroblastoma are the result of traditional two-dimensional culture studies and in vivo models that have been translated to clinical trials. These pre-clinical studies are costly, time consuming, and neglect the study of cofounding factors such as the contributions of the TME. Three-dimensional (3D) bioprinting has become a novel approach to studying adult cancers and is just now incorporating portions of the TME and advancing to study pediatric solid. We review the methods of 3D bioprinting, how researchers have included TME pieces into the prints, and highlight present studies using neuroblastoma. Ultimately, incorporating the elements of the TME that affect neuroblastoma responses to therapy will improve the development of innovative and novel treatments. The use of 3D bioprinting to achieve this aim will prove useful in developing optimal therapies for children with neuroblastoma.

## 1. Introduction

Neuroblastoma, a tumor of neural crest cells, is the most common extracranial solid tumor in children and accounts for 15% of pediatric cancer related deaths [1]. Unlike pediatric hematologic malignancies that have seen remarkable increases in survival and treatment advancements in recent decades, the prognosis for neuroblastoma has not improved dramatically. Lack of major advances in effective therapies may be due, in part, to the tumor microenvironment (TME). Composed of immune cells, mesenchymal cells, stromal cells, and a dense extracellular matrix (ECM), the TME of neuroblastoma creates an unfavorable environment for a variety of therapeutics. Recent studies on the TME have revealed important discoveries, such as *MYCN* being a driver for the immunosuppressive nature of the TME, and further research could prove beneficial to making more efficacious neuroblastoma therapies. Here, we initially characterize the components of the TME hostile to current therapeutics for neuroblastoma, identify the modalities used to gather these findings, and propose the novelty of three-dimensional printing to further advance our understanding of the TME in neuroblastoma.

## 2. The Therapeutic Barriers of the Neuroblastoma Tumor

**Figure 1:**
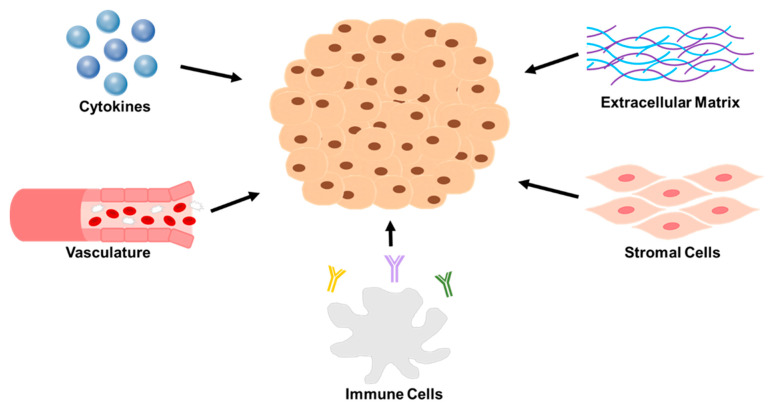
A representation of the five key components of the neuroblastoma TME that pose as major barriers to therapy. Reviewed in this paper are the following for each group. Extracellular matrix: collagen type IV and matrix metalloproteinases. Stromal cells: cancer associated fibroblasts and mesenchymal stromal cells. Immune cells: tumor associated macrophages, myeloid derived suppressor cells, T—lymphocytes, and natural killer cells. Vasculature: angiogenesis factors. Cytokines: VEGF, IL-6, TGF-B, CSF-1, and CXCLs.

Similar to that of the cancer cells themselves, the tumor microenvironment (TME) has its own unique profile on genetic and phenotypic levels. However, the complexity of the TME surmounts that of the tumor cells as it is not limited to a homogenous cellular component nor does it look or behave similar across and amongst malignancies. For example, in breast cancer, the TME promotes brain metastasis [2] while in neuroblastoma it supports the cancer stem cell population [3]. The TME is an intricate network of extracellular matrix (ECM), stromal cells, and immune cells working in a cohesive fashion to foster a favorable home for the residing tumor cells (Figure 1). Individually, these components of the TME fortress have a detrimental toll on the ability to target neuroblastoma by contributing to tumor aggressiveness, posing physical challenges, contributing to chemoresistance and providing a hostile environment to neuroblastoma therapeutics. 

### 2.1. Extracellular Matrix and Stromal Cells

#### 2.1.1. Cancer Associated Fibroblasts

Cancer associated fibroblasts (CAFs) are one of the most abundant cells of the TME and are a key foe in the battle to target neuroblastoma [4]. As a fibroblast derivative, CAFs retain their traditional function contributing to the development of a dense extracellular matrix surrounding tumors and regulating tissue specific extracellular signaling [5], resulting in more aggressive tumors. CAFs are abundant in the neuroblastoma ECM [4]. The development of CAFs is not fully understood, but in other cancers, such as prostate and melanoma, they may be activated through TGFβ signaling and fibroblast activating protein [6,7]. In neuroblastoma, researchers have found that there is an abundance of TGFβ1 in the TME, in part due to the cytokines secreted by CD163+ tumor associated macrophages (TAMs). The cytokine profile produced by TAMs could explain the abundance of CAFs in neuroblastoma [8,9]. Another possible explanation for increased CAFS in neuroblastoma is Schwann cells. The infiltration of Schwann cells into the tumor stroma is a known prognostic determinant for neuroblastoma, with Schwannian poor stroma being associated with worse outcomes [10]. It has been noted that the number of Schwann cells infiltrating the TME is a regulator of CAFs, with a lack of Schwann cells corresponding to an increase in CAFs and their activity [11]. The association of CAFs with more aggressive tumors was corroborated by a study that found stage 4, *MYCN* amplified neuroblastomas had more CAFs, and CAF density significantly correlated with severity of disease [12]. In addition, there is mounting evidence supporting the notion that CAFs contribute to tumor growth in neuroblastoma. It has been hypothesized that the CAF-induced upregulation of JAK/STAT and ERK1/2 signaling may be the responsible mechanism [13]. Collectively, these findings suggest that CAFs in the neuroblastoma TME contribute to tumorigenicity.

CAFs contribute to the evolution of tumor metastasis by stimulating angiogenesis through secretion of vascular endothelial growth factor (VEGF) [11] and altering the ECM through the production of collagen [14]. Collagen is the most abundant macromolecule produced in the CAF-associated ECM of tumors and in an examination of neuroblastoma, collagen type IV was the most predominant type [15]. CAF generated type IV collagen is critical for proper formation of a basement membrane and regulating its structure [5]. In addition to providing scaffolding, these molecules influence angiogenesis and the initial steps to tumor metastasis [16,17]. Collagen IV has also been shown to be critical to neuroblastoma through its effect on integrin binding and cell adhesion. Collagen IV downregulated protein expression of α1β3 integrins and altered levels of matrix metalloproteinases (MMP) 2 and 9 [18]. These MMPs promoted neuroblastoma metastases through ECM destruction, and studies showed that MMP2 was actively secreted and more abundant in the stromal space of highly malignant neuroblastoma [19,20]. Thus, the regulation of collagen production by CAFs is a mainstay to its contribution to neuroblastoma metastasis. 

In addition to overcoming additional oncogenic signaling and increased metastatic potential, CAFs also challenge the success of therapeutics by directly influencing chemoresistance. Scientists have hypothesized that CAFs and the type IV collagen that they produce may lead to a physical barrier to chemotherapies. Yogev et al. found that cyclophosphamide resistant neuroblastoma tumors from a murine model had an increase in the number of CAFs and collagen type IV in the ECM [21]. It has also been postulated that CAF-associated chemoresistance may be due to decreased apoptosis. Borello et al. demonstrated that the coculture of etoposide treated neuroblastoma cells with neuroblastoma specific CAFs, as well as mesenchymal stromal cells (MSCs), led to a decrease in caspase signaling [13]. Investigations with esophageal cancer have shown that CAFs increased the levels of IL-6 in the TME [22]. In neuroblastoma, investigators have shown that IL-6-induced STAT3 signaling may result in neuroblastoma tumor cell evasion of drug-induced apoptosis that could be the due to the effects of CAFs [23]. 

The ECM established by CAFs plays a complex role in the effectiveness of immunotherapy as well. CAFs create a dense ECM that physically hinders infiltrating immunotherapies like CAR-T cells from reaching the tumor cells [24]. Further, the irregular structure and abundance of CAFs within the ECM has been identified as a negative prognostic factor for successful anti-PDL-1 therapy. These findings were based on a pan cancer analysis of TCGA data [25]. The IL-6 produced by CAFs also dampened the effect of immunotherapies by increasing the influx of immunosuppressive T lymphocytes [22].

In summary, CAFs promote tumor aggressiveness, secrete cytokines that result in drug resistance and form a physical barrier to infiltrating immune cells. It is evident that CAFs affect the ability of therapeutics to target neuroblastoma. It is also clear that preclinical evaluations of new therapeutics must consider and include the contributions of CAFs. 

#### 2.1.2. Mesenchymal Stromal Cells

Controversy surrounding the tumorigenic or tumor suppressive activity and duality for use in treatments makes them complex, but several studies have documented that mesenchymal stromal cells have proven problematic for neuroblastoma therapies [26]. Beginning as mesenchymal stem cells in the bone marrow, adipose tissue, and perivascular region of blood vessels, these cells travel to the site of the tumor [27]. Upon arrival, they lose their multipotency and contribute to the cellular component of the TME [28]. The homing of mesenchymal stromal cells has been shown in neuroblastoma. After human mesenchymal stromal cells were administered to *TH-MYCN* transgenic mice, mesenchymal stromal cells were found both in and around the resulting tumors [29]. 

Mesenchymal stromal cells contribute to neuroblastoma progression, metastasis, and chemoresistance [30,31]. Mesenchymal stromal cells have the ability to increase JAK/STAT and ERK 1/2 signaling pathways to promote neuroblastoma growth [13,32]. There is also a cytokine crosstalk between neuroblastoma tumor cells and mesenchymal stromal cells. Together they create a tumorigenic environment through neuroblastoma cells stimulating the secretion of IL-6 and mesenchymal stromal cells secreting VEGF [33,34,35,36]. The ability of mesenchymal stromal cells to promote metastasis also adds another challenge to therapies. An increased number of mesenchymal stromal cells were seen in neuroblastoma bone marrow metastasis [37]. These mesenchymal stromal cells may promote metastasis to the bone marrow through their production of the chemoattractant, CXCL13 [38]. Researchers have shown that mesenchymal stromal cells also contribute to metastasis by supporting the motility and invasiveness of neuroblastoma through a CXCR4 axis by secreting the chemoattractant ligand, SDF-1 (also known as CXCL12) [39,40]. 

The interaction between neuroblastoma cells and mesenchymal stromal cells also leads to resistance to chemotherapies. When neuroblastoma cells were co-cultured with mesenchymal stromal cells, there was an increase in IL-6 levels [23] and subsequent increased activity of STAT signaling that resulted in resistance of the neuroblastoma cells to etoposide [41]. Another reported mechanism is the CXCR4 axis and its interplay between neuroblastoma and mesenchymal stromal cells. Klein et al. showed that overexpression of CXCR4 in SK-N-BE cells resulted in avoidance of apoptotic death from BCL2 inhibitors [32]. Blockade of CXCR4/SDF-1 ligand signaling cascade from mesenchymal stromal cells improved the efficacy of dendritic vaccines in neuroblastoma bearing mice [42]. Additionally, mesenchymal stromal cells alter the effects of immunotherapy for neuroblastoma by limiting antibody dependent cell cytotoxicity (ADCC) through TGFβ signaling. When TGFβ receptor was blocked with CD105 antibody, dinutuximab-induced ADCC was improved in neuroblastoma even in the presence of mesenchymal stromal cells [43]. 

It is important to note that mesenchymal stromal cells may augment immunotherapy. The homing ability of mesenchymal stromal cells has been harnessed to employ these cells as a vehicle to deliver oncolytic viruses [44] or immunostimulant cytokines [45]. Although effective at helping immunotherapies access neuroblastoma tumor sites, evidence presented here suggests that the negative impact of mesenchymal stromal cells may outweigh their benefit. Thus, it is crucial to incorporate mesenchymal stromal cells in the study of neuroblastoma therapeutics because of their potential effects on efficacy. 

#### 2.1.3. Schwann Cells

Schwann cells are a unique contributor to the neuroblastoma TME. Typically, Schwann cells serve as protective glia cells for peripheral nervous system axons. Schwann cell progenitors promote neuroblastoma tumorigenesis by forming into the more malignant, chromaffin-like tumor cells [46]. Contrarily, non-tumor Schwann cells lead to neuroblastoma differentiation. Kwiatkowski compared neuroblastoma cells cultured with media conditioned by Schwann cells to those cultured in media from other stromal components. They found increased tumor cell differentiation in those cells cultured with Schwann cell media [47]. It has been suggested that neuroblastomas expressing NKT1 may recruit Schwann cells to the TME through secretion of neural growth factor and in turn, the migrated Schwann cells will promote differentiation of the tumor [48]. Weiss et al. further show that the Schwann cells in the neuroblastoma TME promoted tumor cell apoptosis, inhibited proliferation, and supported neurite outgrowth (a marker of neuroblastoma differentiation) via epidermal growth factor-like protein 8 [49]. The interaction between neuroblastoma and Schwann cells is not always pro-differentiation, as neuroblastoma may secrete HGMB1 into the TME to induce autophagy of the Schwann cells and ultimately promote tumor proliferation [50]. The complexity of the Schwann cell and neuroblastoma interaction cannot be ignored and should be included in studies with drug therapies targeting neuroblastoma as their potential impact on TME Schwann cells could alter their efficacy.

### 2.2. Immune Cells and Cytokines

#### 2.2.1. Tumor Associated Macrophages

Macrophages are one of the most abundant leukocytes in the TME. As such, macrophages have a major influence on solid tumor progression and their response to therapeutics. Franklin et al. showed using a breast cancer model that the tissue resident macrophages were distinctly different in phenotype, function, and immunoreactivity compared to those macrophages surrounding the tumor [51]. Investigations like these propelled research into tumor associated macrophages (TAMs) and their sophisticated role in neuroblastoma. 

Investigation of neuroblastoma patient samples showed that tumor aggressiveness and metastasis positively correlated with the number of TAMs in the stroma [52]. Subsequent in vitro studies found that peripheral macrophages were converted to TAMs by neuroblastoma conditioned medium. When SK-N-BE(2) cells were co-cultured with TAMs, they demonstrated increased migratory capacity, but their proliferative ability was unaffected. This lack of a direct effect on growth by the TAMs seems contradictory as their presence is increased in rapidly growing and aggressive tumors; however, this study goes on to suggest that TAMs indirectly affect growth through their ability to increase production of cancer promoting CAFs [12]. Other investigators found that the effect of TAMs on tumor growth was more cell line dependent. Hadjidaniel proved that TAMs increased STAT3 signaling in both human (CHLA-255, LAN-6 and LAN-5) and murine neuroblastoma models (NBT2) leading to increased tumor growth [53]. 

The recruitment of TAMs to the TME of neuroblastoma, particularly metastatic lesions, has been attributed to tumor cell secretion of colony-stimulating-factor 1 (CSF-1) [54]. siRNA knockdown of the CSF-1 release by neuroblastoma resulted in decreased tumor progression [55]. In a separate study, CSF-1 receptor blockade on the TAMs prevented their CSF-1-induced attraction and inhibited them from reaching the tumor, which resulted in neuroblastoma tumors becoming more sensitive to cyclophosphamide and topotecan chemotherapies [56]. Not yet shown in neuroblastoma, blockade of CSF-1 in breast cancer also resulted in increased lymphocytic infiltrate. This finding suggested that TAMs could have a negative impact on efficacy of immunotherapies, such as CAR-T cells, by impeding access to the tumor [57]. TAMs in the TME of neuroblastoma also prove problematic to CAR-T cell therapy due to their abundant expression of PDL-1 ligand, blocking T cell activation, and creating an immunosuppressive surrounding [58]. Studies have also found an enrichment of the TAM anti-tumor M2 phenotype [59] in metastatic neuroblastoma sites and that these cells affected the ability of TME natural killer cells to target tumor cells [60]. Mechanisms explaining this effect of TAMs on natural killer cells are not entirely clear, but it is added evidence supporting the need to study neuroblastoma in combination with TAMs.

#### 2.2.2. Myeloid Derived Suppressor Cells

More recently, there have been investigations examining the effects of myeloid derived suppressor cells (MDSCs) in the neuroblastoma TME, particularly regarding their involvement with immunotherapies. As their name suggests, MDSCs lead to immunosuppression in the TME [61]. Researchers have shown that MDSCs are triggered through interaction of the ATP secreted by neuroblastoma with MDSCs’ P2X7R and subsequently release immunosuppressive cytokines [62]. Through polyphenon E modulation of the MDSCs to a more granulocytic phenotype, there was a decrease in these immunosuppressive effects in the neuroblastoma TME [63]. Eliminating the MDSCs with natural killer cells resulted in an improvement of CAR-T cell therapy towards neuroblastoma [64] and improved infiltration of cytotoxic T cells into the tumor [65]. Additionally, in a high-risk neuroblastoma model, the inhibition of MDSCs with BLZ954 resulted in increased efficacy of PDL/PDL-1 blockade [66]. Further research is needed to understand their entire effect on the tumor itself, but it is without question MDSCs are important to study in the context of immunotherapy pre-clinical investigations. 

#### 2.2.3. T Lymphocytes

T lymphocytes are scarce in the TME but their presence correlates with the severity of disease [67,68]. For example, in a study looking at 26 samples of high-risk neuroblastoma, there was a notable amount of CD8+ T cells in the peripheral blood, few within tumor stroma, but none within the tumor itself [69]. The T cells infiltrating the TME often consist of a higher percentage of cytotoxic CD8+ T cells compared to CD4+ helper T cells [70] which prove more cytotoxic to the tumor cell [71]. Clearly increasing the numbers of cytotoxic T cells into the TME could be a successful therapeutic strategy.

Genomic and phenotypic properties of the neuroblastoma tumor itself contribute to the barrier for T cell entry to the TME. As previously described, *MYCN* is an oncogene and is amplified in high-risk neuroblastoma. Analysis of 148 neuroblastomas in the TARGET database revealed that *MYCN* amplified tissue had less T lymphocytic infiltrate as well as other effector immune cells, indicating a lack of inflammatory response to the tumor [72]. Once T cells do infiltrate the TME, whether they are self-procuring or a result of immunotherapy such as CAR-T, the expression of Fas ligand on neuroblastoma cells will induce apoptosis of the T cells and render them useless [73]. Neuroblastoma also impedes T cells through secretion of cytokines. In a Neuro-2-a syngeneic mouse model, the there was an overproduction of macrophage inhibitory factor by the tumor cells that resulted in a deactivation of previously activated T cells [74]. Another mechanism found in neuroblastoma is a lack of monocyte chemoattractant protein-1 (MCP-1) production compared to tumors like medulloblastoma, that have a higher percentage of infiltrating T cells and more MCP-1 [75]. Furthermore, neuroblastoma secrete high mobility group box-1 into the TME which functions to differentiate infiltrating T lymphocytes into immunosuppressive T regulatory cells making the tumor less immunogenic [76]. 

Understanding the complexities of the TME’s effect on T cells is crucial to studying immunotherapy. Researchers have preliminarily shown through co-culture with neuroblastoma that γδ T cells are the optimal lymphocyte subtype for cytotoxicity towards this tumor even in the presence an immunosuppressive TME [77,78]. Furthermore, co-cultured T cells transfected with constitutively active AKT proved resistant to the immunosuppressive neuroblastoma TME [79]. Future studies like these need to be completed to advance CAR-T therapies for use in pediatric solid tumors. 

#### 2.2.4. Natural Killer Cells

Under innate conditions, natural killer cells will attack and respond to cancer cells. In the case of neuroblastoma, natural killer cells are present in the TME but not necessarily active against the tumor, due to the immunosuppressive TME from components already outlined in other discussions in this review. Patients with a low expression of IL-15, an activating cytokine of natural killer cells, and low levels of natural killer cells in the tumor, had poorer outcomes in *MYCN* non-amplified neuroblastoma [80]. 

Once activated, the natural killer cells inhibit neuroblastoma growth, metastasis, and immunosuppression. Early reports of exogenous administration of IL-2, a cytokine known to activate natural killer cells, eliminated neuroblastoma metastasis to the bone marrow [81]. Therapies that take advantage of this finding, have showed that IL-2 in combination with other compounds like IL-18 [82], fractalkine [83], or lenalidomide [84] will ultimately improve immunotherapeutic intervention against neuroblastoma via improved natural killer cell killing. Furthermore, activated natural killer cells release miR-168 exosomes in TME that will ultimately diminish the immunosuppressive effects of neuroblastoma by decreasing MYCN expression and TGF-β release [8]. Thus, understanding the function and presence of natural killer cells in the TME is critical to defining the effects of therapy on neuroblastoma. 

### 2.3. Vasculature

In mouse models [85] and in long term passage neuroblastoma cell lines [20,86], VEGF is the primary mechanism promoting angiogenesis [87]. In examination of 50 neuroblastoma patient samples, vascularity correlated with more aggressive tumors [88]. Multiple methods to inhibit angiogenesis, including decreased notch signaling [89], prostaglandin E synthase inhibitors [90], targeting hypoxia-inducible factors [91], or limiting infiltrating pericytes [92], have been shown to hinder neuroblastoma tumor progression and metastasis. The pro-angiogenic factors expressed and released by neuroblastoma also contribute to alterations in the extracellular matrix, adding complexity to therapeutic intervention [93]. Haagendoorn and colleagues showed that vasculature of solid tumors is irregular and results in difficult drug delivery [94]. Therefore, when given bevacizumab to block VEGF, it not only altered the tumor morphology but improved vessel function and delivery of chemotherapeutics to neuroblastoma xenografts by noticeably decreasing tumor size [95]. This supports the need to incorporate the understanding of tumor vasculature in pre-clinical testing of neuroblastoma therapy. 

## 3. Three-Dimensional Bioprinting: Applications to Neuroblastoma

### 3.1. Three-Dimensional Models

Three-dimensional models are being utilized more frequently for cancer studies due to their ability to create tissue-like structures more effectively than monolayer cell cultures. The main limitation to two-dimensional cell culture is the inability of those models to imitate the in vivo architecture and microenvironments [96]. A group at the Lawrence Berkeley National Laboratory demonstrated the ability of three-dimensional models to outperform two-dimensional models. This group showed that in a three-dimensional breast cancer model, the antibodies against the cell-surface receptor 1-integrin changed the behavior of cancerous breast cells such that they become non-cancerous, losing their shapes and patterns of growth. This alteration of shape and functionality of the breast cancer cells was not observed in the two-dimensional model [97]. 

Spherical cancer models are the most commonly used in vitro three-dimensional model in cancer research. There are four groups of spherical cancer models including multicellular tumor spheroids, tumorspheres, tissue-derived tumor spheres, and organotypic multicellular spheroids [98]. Multicellular tumor spheroid models are generated from single-cell suspension cultures, with the incorporation of fetal bovine serum and no externally supplied extracellular matrix. These models establish the functional and morphologic properties of tissue in vivo [99]. The single-cell suspension cell cultures used for multicellular tumor spheroid models typically originate from permanent cancer cell lines, and do not commonly utilize cells from dissociated tumor tissue [98]. Free-floating cancer stem cell spheres, also known as tumorspheres, were initially described in brain tumor research [100]. Since their initial discovery, they have been developed from a large range of solid tumors, including breast [101], pancreatic [102], and ovarian cancers [103]. It has been observed that tumorspheres do not fully recapitulate the three-dimensional environment and structure of an in vivo tumor. Tumorspheres are typically used as models to study cancer stem cell properties rather than models for mimicking cancer tissues [104]. The third category of spherical cancer models are tissue-derived tumor spheres, which are derived from partially dissociated cancerous tissues [98]. This spherical model has been mostly employed in colorectal cancer research [105]. While tissue-derived tumor spheres typically recapitulate avascular tumor microregions, the major downfall of this spherical model is the lack of cell proliferation seen in vivo [98]. The final category of spherical models is organotypic multicellular spheroids, constructed from culturing ex vivo fragments of tumors without dissociation [106]. This spherical model has been generated from several tumor types including glioblastoma [107], meningioma [108], mesothelioma [109], and colorectal cancer [110]. As compared to the other three types of spherical models described, organotypic multicellular spheroids most closely model in vivo tumors by best recapitulating the native tumor heterogeneity, with the downfall of this model being the limited types of cancer in which it has been reported [98]. 

### 3.2. Three-Dimensional Bioprinting

A technique that allows for the creation of three-dimensional cellular constructs involves the utilization of a three-dimensional bioprinter. A three-dimensional bioprinter allows for fabrication in the X, Y, and Z directions as created from computer-aided design software or scanned from medical images [111]. Bioprinting is performed through layered deposition of bioink in a spatially defined manner. Bioink is a hydrogel-based biomaterial solution that is used to create the tissue constructs. The biomaterials used to create hydrogel bioink include, but are not limited to, alginate, gelatin, collagen, hyaluronic acid, and agarose [112]. Hydrogel-based bioinks are regularly used as they are biocompatible and typically capable of aiding in cell attachment and differentiation [113]. The main disadvantage hydrogel bioinks is their lack of mechanical strength [114]. To compensate for this lack of mechanical strength, the structures may be cross-linked to obtain a more secure structure [115]. 

The bioink that is best suited for the specific model that is being researched will possess the desired physiochemical properties of the tissue in question, including chemical and biological characteristics [116]. For example, successful bioprinted models of breast and pancreatic cancer were created using a reversibly cross-linkable hydrogel bioink, composed of 1% Pronova Ultrapure Low Viscosity Sodium Alginate and 6% gelatin in phosphate buffered saline (PBS). Fibroblasts, endothelial cells, additional stromal cells, and growth factors were also used in these models to help create the physiochemical properties of the cancer in question. This group further demonstrated that multi-cell-type bioprinted tissues were able to recapitulate the TME of in vivo neoplastic tissues [117].

The primary three-dimensional bioprinting techniques used are inkjet-based, extrusion-based, and laser-assisted printing [118]. Inkjet-based printers distribute precise picoliters of bioink [119]. The main limitation to inkjet-based bioprinting is the difficulty in obtaining biologically relevant cell densities [120]. Some advantages of inkjet-based bioprinting include its low-cost, high speed, and biocompatibility with many materials [121]. The most notable applications of inkjet-based bioprinting are regeneration of skin [122] and cartilage [123]. Extrusion-based bioprinting uses pneumatic or mechanical dispensing systems for the continuous extrusion of biomaterials [118]. This form of bioprinting is advantageous as it has the capacity to deposit large cell densities [121]. This technique has been shown to be useful in cancer research. Xu et al. utilized an extrusion-based bioprinter for high-throughput drug screening in ovarian cancer, in which they investigated regulatory feedback mechanisms in vitro [124]. Laser-assisted bioprinting utilizes laser-induced forward-transfer to generate scaffold-free three-dimensional systems via layered deposition of bioink [125]. This method of bioprinting is typically used for applications in tissue and organ engineering and is known for its high cell viability [126].

Bioprinting allows for high-throughput, automated control of structures that have high reproducibility [112]. An example of the high-throughput application of three-dimensional bioprinting to cancer models was demonstrated by the creation of three-dimensional breast epithelial spheroids. Swaminathan and colleagues demonstrated that a three-dimensional bioprinter had the capacity to create multicellular breast tumor spheroids while simultaneously maintaining the function, structure, and polarization of the spheroids. These spheroids were then immediately used for assays such as drug screening [127]. This automation and high-throughput capacity of three-dimensional printing is especially enticing in cancer research as it bridges the gap that currently exists between cancer models and preclinical trials. It is standard for preclinical trials to be based on animal models, with patient-derived tumor xenografts typically used to recreate the native tumor heterogeneity and test cancer therapeutics [128]. Bioprinting three-dimensional cancer models serves as a bridge between animal experimentation and human trials [129]. 

### 3.3. Recapitulation of the Tumor Microenvironment with Three-Dimensional Bioprinting

A major challenge in the creation of cancer models is the recapitulation of the native tumor microenvironment (TME). Two-dimensional culture systems do not contain the architectural structure and microenvironment of the tumor [130]. Three-dimensional models of cancer have shown to better represent the physiological conditions as compared to traditional two-dimensional models, by recapitulating the native TME and spatial distribution of cells [131]. Amongst the three-dimensional models utilized, spherical models have shown the most significant promise for creating the appropriate microenvironment [132]. Three-dimensional spherical cell culture models allow for the cell–cell and cell-extracellular matrix interactions that are necessary to mimic the native TME [133]. Three-dimensional constructs that contain patient-derived cells may be propagated in vitro to mimic the native TME that exists in vivo [132]. As previously discussed, three-dimensional bioprinting is an ideal platform to create three-dimensional constructs, and therefore maybe used to model the TME. The incorporation of various cell types such as cancer associated fibroblasts, mesenchymal stromal cells, tumor associated macrophages, and functional vasculature into the bioink can assist in the recapitulation of the native TME in three-dimensional bioprints. 

It has been demonstrated that three-dimensional bioprinting that incorporated cancer-associated fibroblasts allowed for the recreation of the native TME by Langer et al. For their breast tumor study, the estrogen receptor-positive MCF-7 cell line was bioprinted with the incorporation of primary human mammary fibroblasts and human umbilical vein endothelial cells in the bioink. Immunofluorescence staining of the bioprints displayed the interaction between epithelial cancer cells and stromal fibroblasts. This study then went on to demonstrate that distinct microenvironments could be modeled using bioprinted tissues. The investigators included primary subcutaneous preadipocytes into the tissue stromal compartment along with human mammary fibroblasts and human umbilical vein endothelial cells. They successfully showed that additional tissue-relevant cell types could be included in the bioprints to successfully recapitulate the native TME. These results were proven by quantifying the adipocyte maturation within the bioprints over a course of ten days post-printing [117].

The inclusion of mesenchymal stromal cells into three-dimensional bioprints has also shown potential for recapitulating the native TME. This method was detailed by Byambaa et al. in their bioprinting of three-dimensional bone tissue. In this study, the bioprinted bone tissue constructs acted as biomimetic in vitro matrices capable of coculturing bone marrow derived mesenchymal stem cells and human umbilical vein endothelial cells. Through immunostaining and RT-qPCR, it was confirmed that the encapsulated human mesenchymal stem cells were able to form a mature bone marrow niche after 21 days of culture. The bioprinting method described in this study demonstrated the control of local physical and chemical microniches and established gradients in the bioprinted constructs [134]. While this study focused on the engineering of bone tissue, the applications of the effects of using mesenchymal stromal cells in three-dimensional bioprints could be extended beyond bone tissue and applied to cancer models. Mesenchymal stromal cells have also been successfully incorporated into three-dimensional bioprints of cartilage, cardiovascular tissue, neural tissues, and tendons, thus speaking to their ability to enhance the TME of bioprints [135].

Tumor associated macrophages also have the capacity to enhance the TME of three-dimensional bioprinted tissues. Tang et al. demonstrated the use of macrophages in the establishment and proliferation of three-dimensionally bioprinted glioblastoma models. One aspect of this study focused on the hypoxic and invasive signatures of the bioprinted models of glioblastoma. Bioprinted cultures containing the human monocytic cell line, THP-1, derived macrophages, human induced pluripotent stem cell-derived macrophages and primary human volunteer-derived macrophages were created, and RNA-seq was performed on the models to understand the relative contributions of gene expression for each cell type in the bioprinted model. This study showed that the THP-1 derived macrophages promoted the hypoxic and invasive signatures of the bioprinted models. Ultimately, the results of this study demonstrated the critical nature of growth factor signaling elements that are provided from immune fractions, such as macrophages, to the brain tumor model [136]. 

A common issue with three-dimensionally printing tissue is the difficulty in creating functional vasculature within the tissue [137]. For large tissue constructs, vascularization of the tissue is critical for maintaining the tissue’s viability [138]. Zhang et al. demonstrated the ability to use three-dimensional printing to create a vasculature network directly by bioprinting vessel-like cellular microfluidic channels using hydrogels [139]. Thus, as vascularization is a necessity in maintaining the viability of tissues, incorporating vascularization into three-dimensional prints can assist in the recapitulation of the TME. 

### 3.4. Three-Dimensional Bioprinting in Neuroblastoma

There is literature supporting the use of three-dimensional bioprinting in creating in vitro models of neuroblastoma. The foundation of these studies is provided by the three-dimensional in vitro models of neuroblastoma that have been used for pre-clinical assessments. Three-dimensional models of neuroblastoma have been observed in several in vitro culture systems including multicellular tumor spheroids [140], tissue-derived tumor spheres [141], and patient-derived tumor organoids [142]. Based on the success of these three-dimensional models and the ability of bioprinters to create three-dimensional models, it is logical that a three-dimensional bioprinter would be a tool of significant value in creating three-dimensional neuroblastoma models. Promising initial results have been seen in the application of three-dimensional bioprinting to neuroblastoma cell lines. There are data that establish the bioprinting protocol of the neuroblastoma cell line SH-SY5Y, which allowed for cell viability to be maintained after five days of culture. This study demonstrated that the neural cells organized themselves into distinct colonies in the three-dimensional environment, thereby confirming that the three-dimensional structure created by the protocol was optimal based on the proposed bioink [143]. There are also data that detail the creation of three-dimensionally printed scaffolds using SH-SY5Y neuroblastoma cells. This study ultimately demonstrated the role of three-dimensional bioprinting in the creation of a realistic in vitro three-dimensional neural model using a neuroblastoma cell line [144]. There is extensive potential for three-dimensional bioprinting applications and techniques to be expanded upon, and the literature reviewed demonstrates the capacity for three-dimensional bioprinting to create reliable neuroblastoma models for cancer research.

## 4. Conclusions

**Figure 2:**
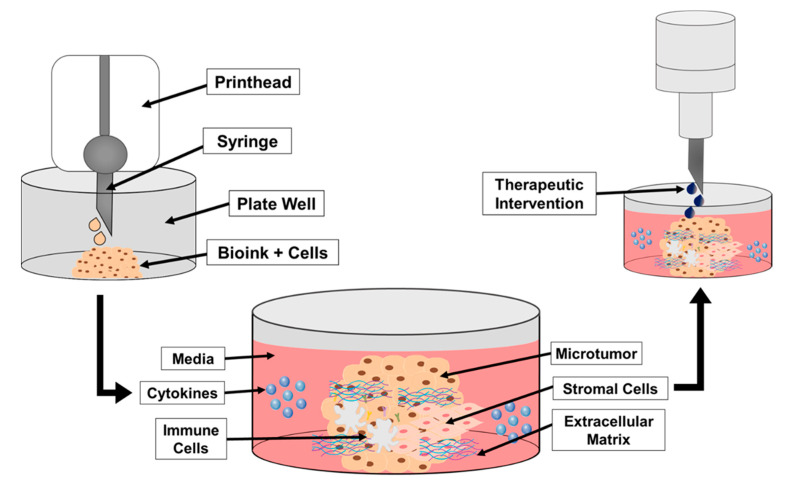
A schematic demonstrating the steps in bioprinting neuroblastoma cells, TME cells and components, and addition of cytokines. Once established, treatments may be added to the print to assess therapeutic response and effectiveness better than a 2D cell culture. The initial step in Figure 2 demonstrates the bioprinting of the bioink and tumor cell mixture into the designated well plate. The printed structure is then cross-linked for structural support and the media is added to the well. From there, additional components representing the TME may be added to the print and surrounding media to better mimic the TME (e.g., cytokines, immune cells, stromal cells). The final step in Figure 2 shows the addition of the therapeutic intervention on the printed structure with TME components.

The neuroblastoma TME has the potential to contribute to tumor aggressiveness and therapeutic resistance. Novel 3D bioprinting shows promise for the study of the TME in cancer. Currently, researchers have been successful in using 3D printing to create solid tumors, including neuroblastoma (Table 1). Supplementing the prints with TME components could provide the means to better investigate potential chemotherapeutics in a pre-clinical setting (Figure 2). As our knowledge of neuroblastoma grows more robust, the evolution of neuroblastoma investigations will progress and will likely include the incorporation of artificial tumor microenvironments. 

## Figures and Tables

**Table 1:** Three-Dimensional Bioprinting in Cancer. Different cancers and their respective bioprinting methods reviewed for this paper.

Type of Three- Dimensional Printer	Printing Method	Cancer Tissues Printed	Reference
Inkjet Based	Fast distribution of bioink droplets	Bladder	[140]
		Breast	[141]
Extrusion Based	Slow continuous distribution of bioink	Neuroblastoma	[138]
		Breast	[112]
		Pancreatic	[112]
Laser Assisted	Medium-fast laser-induced forward-transfer of bioink	Pancreatic	[142]

## Data Availability

Data sharing not applicable. We did not create or analyze new data in this study.
